# Exosomal hsa_circ_0000519 modulates the NSCLC cell growth and metastasis via miR-1258/RHOV axis

**DOI:** 10.1515/med-2022-0428

**Published:** 2022-04-28

**Authors:** Rui Wang, Hongliu Liu, Mingqiang Dong, Dan Huang, Jun Yi

**Affiliations:** Department of Oncology, Jingmen No. 1 People’s Hospital, Jingmen, Hubei, China; Department of Health Care for Cadres, Jingmen No. 1 People’s Hospital, Jingmen, Hubei, China; Department of Cardiothoracic Surgery, Jingmen No. 1 People’s Hospital, Jingmen, Hubei, China

**Keywords:** non-small cell lung cancer, exosome, hsa_circ_0000519, miR-1258, RHOV

## Abstract

This study aims to explore the function and mechanism of exosomal circ_0000519 in non-small cell lung cancer (NSCLC) development. Expression of circ_0000519, microRNA (miR)-1258, and Ras homolog gene family V (RHOV) in serum samples of NSCLC patients or cell lines were examined via quantitative reverse transcription-polymerase chain reaction and Western blotting. The function of circ_0000519 was evaluated through 5-ethynyl-2′-deoxyuridine (EdU) staining, colony formation, transwell, Western blotting, xenograft, and immunohistochemistry analyses. The binding relationship was evaluated by a dual-luciferase reporter assay and RNA pull-down assay. Results showed that circ_0000519 abundance was enhanced in the serum samples of NSCLC patients and cells. circ_0000519 knockdown suppressed the cell growth by decreasing the colony-formation ability and Cyclin D1 expression and inhibited cell metastasis via reducing migration, invasion, and levels of Vimentin and matrix metalloproteinase 9 (MMP9). circ_0000519 overexpression promoted cell growth and metastasis. circ_0000519 was carried by exosomes and knockdown of exosomal circ_0000519 suppressed the cell growth and metastasis. miR-1258 was downregulated in NSCLC cells and targeted by circ_0000519. RHOV was targeted by miR-1258 and upregulated in the NSCLC cells. miR-1258 knockdown or RHOV overexpression attenuated the influence of exosomal circ_0000519 knockdown on cell growth and metastasis. Exosomal circ_0000519 knockdown decreased xenograft tumor growth. Collectively, the knockdown of exosomal circ_0000519 repressed the cell growth and metastasis in NSCLC through the miR-1258/RHOV axis, which provided a new insight into NSCLC development and treatment.

## 1 Introduction

Lung cancer is a frequent tumor with 2.09 million newly diagnosed cases and 1.76 million deaths in 2018 globally [1]. Non-small cell lung cancer (NSCLC) is the major histological subtype of lung cancer accounting for ∼85% of cases, including lung adenocarcinoma, squamous cell carcinoma, and lung large cell carcinoma [2]. Significant progress has been gained in the diagnosis and treatment of NSCLC, but the 60-month survival of patients is poor, especially in the advanced stage [3]. Hence, new therapeutic strategies are needed to improve the outcomes of NSCLC.

Exosomes are single-membrane vesicles with a diameter of ∼30 to ∼200 nm, which can be secreted by most cells, and take part in cell-to-cell communication in human health and diseases [4,5]. Exosomes can transfer proteins, DNA, mRNA, and non-coding RNAs, which have important roles in tumor growth, metastasis, and angiogenesis [6,7]. The tumor-derived exosomes can be secreted in the tumor environment, thus affecting cancer growth, progression, metastasis, and drug resistance in NSCLC [8]. As reported, dendritic cell-derived exosomes can receive immune signals and use a cell-free vaccine for cancer immunotherapy [9]. Circular RNAs (circRNAs) are a type of important non-coding RNAs, which can be enriched in exosomes and dysregulated in human diseases, especially in cancers [10]. Moreover, circRNAs can participate in NSCLC progression via modulating microRNA (miRNA)/mRNA networks [11]. Additionally, exosomal circRNAs can regulate NSCLC growth, metastasis, and glycolysis, such as circRNA Rho GTPase-activating protein 10 (ARHGAP10) and mediator of cell motility 1 (MEMO1) [12,13]. A previous report founds that hsa_circ_0000519 (circ_0000519, also named hsa_circ_002178) is an upregulated circRNA in lung adenocarcinoma, as revealed by the GSE101684 and GSE101586 datasets, and it can be transferred by exosomes to be involved in immune therapy by regulating programmed death-ligand 1 (PDL1)/programmed cell death protein 1 (PD1) [14]. But, the exact role and mechanism of exosomal circ_0000519 in NSCLC development are unknown.

MiRNAs are short non-coding RNAs that regulate various processes such as growth, apoptosis, metastasis, differentiation, and metabolism, which have key roles in the progression, diagnosis, and therapy of NSCLC [15,16]. miR-1258 is a miRNA associated with the heparinase expression and suppresses cell invasion in NSCLC [17]. Furthermore, miR-1258 can inhibit cell proliferation and promote apoptosis in NSCLC by regulating growth-factor-receptor-bound protein 2 (GRB2) [18]. Nevertheless, whether the miRNA is regulated by circ_0000519 remains unknown. Additionally, Ras homolog gene family V (RHOV) is an overexpressed gene in NSCLC [19]. And, it may be related to the poor prognosis of NSCLC [20]. However, the function of RHOV in NSCLC has not been reported. The bioinformatics analysis predicts that both circ_0000519 and RHOV can bind with miR-1258, suggesting the potential competitive mechanism among the three. Yet, no study has clarified the network of circ_0000519/miR-1258/RHOV in NSCLC.

The purposes of our study were to analyze the function of exosomal circ_0000519 on NSCLC growth and metastasis and to confirm the network of circ_0000519/miR-1258/RHOV in NSCLC progression. This study might provide a novel mechanism for understanding the pathogenesis of NSCLC.

## 2 Methods

### 2.1 Bioinformatics analysis

The information of circ_0000519 was provided by circBase (http://circrna.org/). The downstream miRNAs of circ_0000519 were searched from circinteractome (https://circinteractome.nia.nih.gov/). The target mRNAs of miR-1258 were searched from TargetScan (http://www.targetscan.org/vert_71/).

### 2.2 Clinical samples

The serum samples were harvested from NSCLC patients (*n* = 59) in Jingmen No.1 People’s Hospital. Age- and gender-matched normal volunteers (*n* = 37) were used as controls.


**Ethical approval:** The present study was approved by the ethical review committee of Jingmen No. 1 People’s Hospital. Written informed consent was obtained from all enrolled patients.
**Informed consent:** Patients agree to participate in this work.

### 2.3 Cell culture

Lung squamous cell carcinoma cell line (H2170 cells), lung large cell carcinoma cell line (H1299 cells), and lung adenocarcinoma cell line (A549 cells) were purchased from Procell (Wuhan, China) and cultured in Roswell Park Memorial Institute (RPMI)-1640 medium (Gibco, Grand Island, NY, USA) plus 10% fetal bovine serum (FBS) (Gibco) and 1% penicillin/streptomycin (Gibco) at 37°C and 5% CO_2_. Human bronchial epithelioid cell line (BEAS-2B cells) was purchased from Procell and cultured in the specific Bronchial Epithelial Cell Growth Medium (Lonza, Hayward, CA, USA) at 37°C and 5% CO_2_.

### 2.4 RNase R and Actinomycin D assays

The circular structure of circ_0000519 was analyzed via RNase R and Actinomycin D assays. For the Actinomycin D assay, H2170, H1299, and A549 cells were incubated with 2 μg/mL Actinomycin D (Sigma, St. Louis, MO, USA) for different time points. Next, the cells were lysed for RNA isolation, and abundances of circ_0000519 and GAPDH were measured.

For RNase R assay, total RNA was exposed to 2 U/μg RNase R (Geneseed, Guangzhou, China) for 20 min and next used for the detection of circ_0000519 and GAPDH levels.

### 2.5 Cell transfection

The circ_0000519 overexpression vector was constructed via Geneseed, and the pCD5-ciR vector was regarded as a negative control (vector). RHOV overexpression vector (pcDNA3.1-RHOV) was constructed in our laboratory with a pcDNA3.1 vector (Thermo Fisher Scientific, Waltham, MA, USA) alone as a negative control. The small-interfering RNA (siRNA) for circ_0000519 (si-circ_0000519), siRNA negative control (si-NC), miR-1258 mimic, negative control of mimic (miR-NC), miR-1258 inhibitor (anti-miR-1258), and inhibitor negative control (anti-NC) were generated via Genomeditech (Shanghai, China). The oligonucleotide sequences are listed in Table 1. H2170, H1299, or A549 cells were incubated with 2 μg vectors or 20 nM oligonucleotides and 5 μL Lipofectamine 3000 (Thermo Fisher Scientific) for 12 h. Cells were harvested for further studies after 24 h post-transfection.

**Table 1:** The oligo sequences for transfection in this study

Name	Sequence (5′–3′)
si-circ_0000519	ACGCACUCAGCUCCCCGAAGC
si-NC	UGCGUGAGUCCUCCCCGAAGC
miR-1258 mimic	AGUUAGGAUUAGGUCGUGGAA
miR-NC	CGAUCGCAUCAGCAUCGAUUGC
anti-miR-1258	UUCCACGACCUAAUCCUAACU
anti-NC	UGAGCUGCAUAGAGUAGUGAUUA

### 2.6 Quantitative reverse transcription-polymerase chain reaction (qRT-PCR)

Total RNA was extracted with Trizol (Thermo Fisher Scientific) or an exosome RNA isolation kit (Norgen, Thorold, Canada) and reversely transcribed to cDNA using a miScript reverse transcription kit (Qiagen, Dusseldorf, Germany) or M-MLV Reverse Transcriptase kit (Thermo Fisher Scientific). The generated cDNA was diluted 10 times and used for qRT-PCR after being mixed with SYBR (Thermo Fisher Scientific) and specific primer pairs (Sangon, Shanghai, China). The qRT-PCR was conducted on the CFX96™ Real-time PCR Detection System (Bio-Rad, Hercules, CA, USA). The primer sequences are designed and listed in Table 2. GAPDH (for circRNA or mRNA) and U6 (for miRNA) acted as controls. The relative RNA level was measured according to the 2^−ΔΔCt^ method.

**Table 2:** The primer sequences for qRT-PCR in this study

Name	Sequence (5′–3′)	
	Forward	Reverse
miR-1258	GCCGAGAGTTAGGATTAGGTC	AGTGCAGGGTCCGAGGTATT
U6	CTCGCTTCGGCAGCACA	AACGCTTCACGAATTTGCGT
circ_0000519	GCCCTAACAGGGCTCTCC	CAGACCTTCCCAAGGGACAT
RHOV	CAGTCACCTCCGAGCAGTTT	TCTCCAAATCGGGGTTGAGC
GAPDH	AATGGGCAGCCGTTAGGAAA	GCGCCCAATACGACCAAATC

### 2.7 5-Ethynyl-2′-deoxyuridine (EdU) staining

Cell proliferation was analyzed with a BeyoClick™ EdU Cell Proliferation Kit with Alexa Fluor 594 (Beyotime, Shanghai, China). In brief, 4 × 10^4^ H1299, H2170, or A549 cells were added to 24-well plates and incubated for 24 h. Next, the cells were interacted with 10 μM EdU for 2 h and treated with 4% paraformaldehyde (Beyotime) and 0.3% Triton X-100 (Beyotime), followed by incubating with the click reaction solution for 30 min. The nuclei were stained with 4′,6-diamidino-2-phenylindole (DAPI; Beyotime) for 10 min. The Edu-positive cells were observed with a fluorescence microscope (Olympus, Tokyo, Japan).

### 2.8 Colony formation assay

Cell growth was assessed via a colony formation assay. Briefly, 800 H2170, H1299, or A549 cells were added to 6-well plates. After 10 days of culture, the colonies were stained using 0.1% crystal violet (Solarbio, Beijing, China) followed by taking pictures and calculating.

### 2.9 Transwell analysis

Cell migration and invasion were analyzed with 24-well transwell chambers (Corning Inc., Corning, NY, USA). In brief, for the migration assay, 1 × 10^5^ H2170, H1299, or A549 cells were resuspended in the non-serum RPMI-1640 medium and added to the upper chambers. The lower chambers were filled with RPMI-1640 medium plus 10% FBS as a chemoattractant. After a culture of 24 h, the cells in the lower chambers were dyed using 0.1% crystal violet and observed under a microscope (100× magnification; Olympus). For invasion analysis, the transwell chambers were pre-coated with Matrigel (Solarbio), and 5 × 10^5^ H2170, H1299, or A549 cells were added to the upper chambers. Other protocols were the same as the migration assay. The migratory or invasive number of cells was analyzed using Image J v1.6 (NIH, Bethesda, MD, USA).

### 2.10 Western blotting

The protein was extracted using radioimmunoprecipitation assay buffer (Thermo Fisher Scientific). The samples were quantified using a bicinchoninic acid kit (Thermo Fisher Scientific), and 20 μg of samples were separated via sodium dodecyl sulfate-polyacrylamide gel electrophoresis and transferred on polyvinylidene fluoride membranes (Bio-Rad). The membranes were incubated in 3% bovine serum albumin for 1 h and next interacted with the primary antibodies Cyclin D1 (ab226977, 1:500 dilution, Abcam, Cambridge, UK), Vimentin (ab137321, 1:300 dilution, Abcam), matrix metalloproteinase 9 (MMP9) (ab76003, 1:3,000 dilution, Abcam), cluster of differentiation (CD) 63 (ab216130, 1:200 dilution, Abcam), CD81 (ab109201, 1:2,000 dilution, Abcam), RHOV (sc-515072, 1:500 dilution, Santa Cruz Biotechnology, Santa Cruz, CA, USA), and GAPDH (ab181603, 1:5,000 dilution, Abcam) overnight. GAPDH functioned as a normalized control. The blots were exposed to enhanced chemiluminescence (Beyotime) and evaluated with Image J v1.6.

### 2.11 Exosome isolation, validation, and incubation

After culturing for 48 h, the culture medium of H1299 cells was collected and centrifugated at 3,000×*g* for 15 min, followed via the use of exosome isolation with an exosome isolation kit (Thermo Fisher Scientific) following the instructions. To inhibit the secretion of exosomes, H1299 cells were pre-treated with 20 μM GW4869 (Sigma) for 2 h before exosome isolation. The exosome structure was confirmed via transmission electron microscope (TEM; JEOL, Tokyo, Japan). The particle size and concentration of the exosome were analyzed using ZetaView PMX 110 (Particle Metrix, Meerbusch, Germany). The specific exosomal markers CD63 and CD81 were measured by Western blotting. To investigate the role of exosomes, H2170 and A549 cells were incubated with 5 μg/mL exosomes for 24 h.

### 2.12 Dual-luciferase reporter and RNA pull-down assays

The wild-type (WT) sequence of circ_0000519 or RHOV 3′UTR with miR-1258 binding sites was cloned into the pmir-GLO vector (Promega, Madison, WI, USA), generating the circ_0000519 WT and RHOV 3′UTR WT vectors. The mutant (MUT) sequence containing the mutant sites was used to construct the circ_0000519 MUT and RHOV 3′UTR MUT vectors. The constructed vectors were co-transfected with miR-1258 mimic or miR-NC into H2170, H1299, or A549 cells with Lipofectamine 3000. After 24 h, luciferase activity was determined through a dual-luciferase reporter assay kit (Promega) following the instructions.

The RNA pull-down analysis was conducted with a Pierce™ Magnetic RNA-Protein Pull-Down Kit (Thermo Fisher Scientific) following the instructions. In brief, the biotin-labeled miR-1258 (Bio-miR-1258) or Bio-miR-NC was generated by the RNA 3′ end desthiobiotinylation kit (Thermo Fisher Scientific) and transfected into H2170, H1299 or A549 cells for 24 h. Next, 1 × 10^7^ cells were lysed and interacted with the streptavidin magnetic beads for 6 h. Then, enriched RNA was eluted, and circ_0000519 level was measured via qRT-PCR.

### 2.13 Xenograft model

The lentivirus-carrying short hairpin RNA (shRNA) for circ_0000519 (sh-circ_0000519) or negative control (sh-NC) was constructed by Genomeditech and transfected into H1299 cells according to the instructions. The exosomes were isolated from the transfected cells and named sh-NC-exo or sh-circ_0000519-exo. Five-week-old male BALB/c nude mice (Vital River, Beijing, China) were divided into two groups (*n* = 5/group), and subcutaneously injected with 5 × 10^6^ H2170 cells. After 5 days, the mice were injected with 30 μg sh-NC-exo or sh-circ_0000519-exo twice a week at the tumor sites. Tumor volume was detected every week, and calculated with the formula (0.5 × length × width^2^). No unexpected deaths occurred during the study. After 30 days, mice were euthanized through inhalation anesthesia of 5% isoflurane (Sigma). Then tumors were harvested and weighed. circ_0000519, miR-1258, and RHOV levels in the tumors were detected. The procedures were permitted via the Animal Ethics Committee of Jingmen No.1 People’s Hospital.

### 2.14 Immunohistochemistry

The tumors were sectioned to 4 μm thickness, and the sections were blocked by 3% H_2_O_2_. The sections interacted with the primary antibody for Ki-67 (ab15580, 1:300 dilution, Abcam) overnight and horseradish peroxidase-labeled IgG (ab6721, 1:1,000 dilution, Abcam) for 2 h, followed by staining with diaminobenzidine (DAB; Beyotime). The sections were observed under a microscope.

### 2.15 Statistical analysis

The experiments were conducted three times. Data were shown as mean ± standard deviation (SD). The linear correlation of RNA levels in NSCLC patients was analyzed by the Pearson coefficient test. The difference was compared via Student *t*-test or one-way analysis of variance with the Tukey *post hoc* test, which was processed through GraphPad Prism 8 (GraphPad Inc., La Jolla, CA, USA). *P* < 0.05 predicted the significant difference.

## 3 Results

### 3.1 circ_0000519 level is elevated in NSCLC

**Figure 1:**
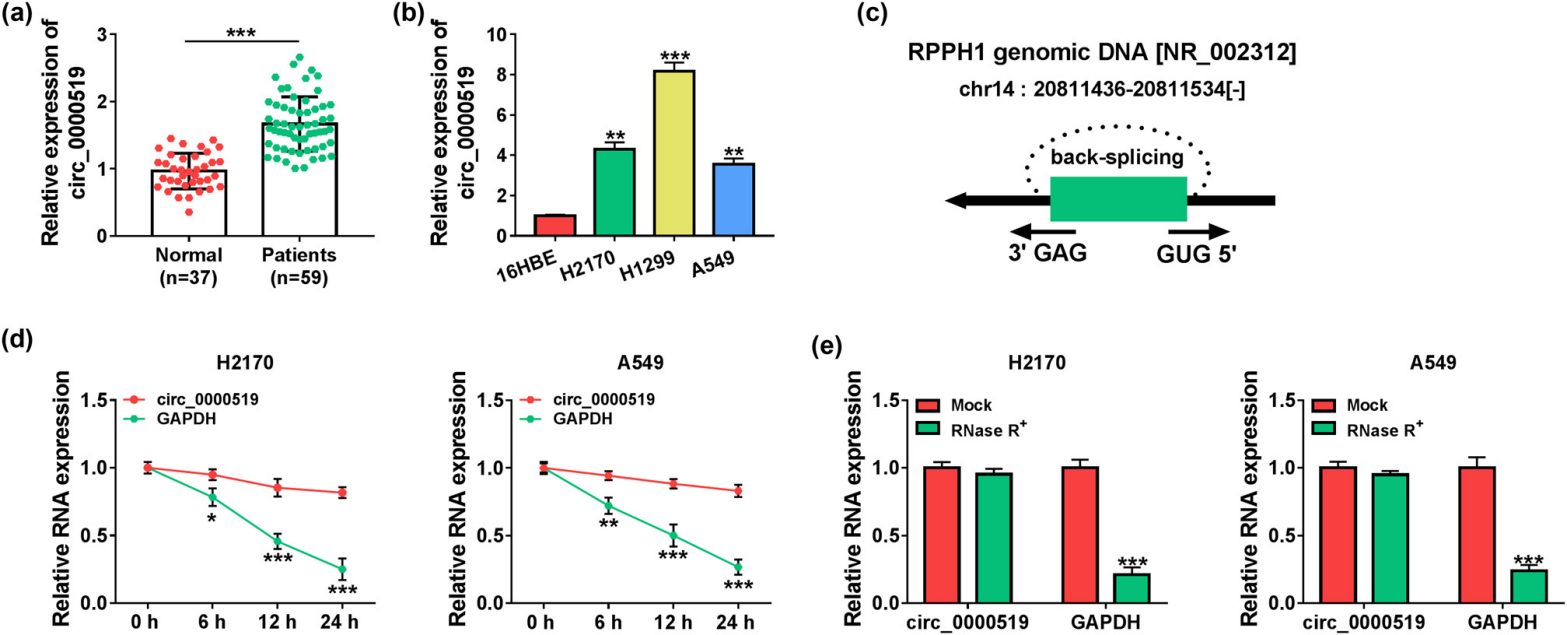
circ_0000519 level is increased in NSCLC. (a) circ_0000519 level was measured via qRT-PCR in the serum samples of NSCLC patients (*n* = 59) or normal subjects (*n* = 37). (b) circ_0000519 abundance was detected via qRT-PCR in H2170, H1299, A549, and BEAS-2B cells. (c) The information of circ_0000519 was searched from circBase. (d) circ_0000519 and GAPDH levels were measured by qRT-PCR in H2170 and A549 cells after exposure to Actinomycin D for different time points. (e) circ_0000519 and GAPDH abundances were determined via qRT-PCR in H2170 and A549 cells after the incubation of RNase R. ^*^ *P* < 0.05, ^**^ *P* < 0.01, and ^***^ *P* < 0.001.

**Figure A1:**
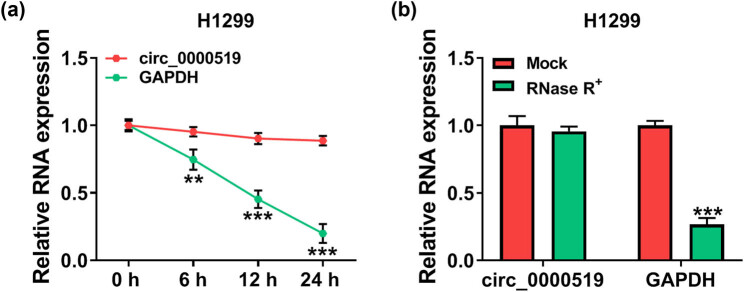
The circular structure of circ_0000519 was identified by Actinomycin D and RNase R treatment assays. ^**^P < 0.01, and ^***^P < 0.001.

To determine if circ_0000519 was involved in the NSCLC development, circ_0000519 expression was detected in NSCLC patients. The serum samples were harvested from 59 NSCLC patients and 37 normal subjects. circ_0000519 level was higher in the NSCLC patients than in the normal group (Figure 1a). Moreover, circ_0000519 abundance was enhanced in the H2170, H1299, and A549 cells when compared with the BEAS-2B cells (Figure 1b). The information of circ_0000519 was obtained from the circBase database, which showed that circ_0000519 was derived from the ribonuclease *P* RNA component H1 (RPPH1) gene at chr14: 20811436-20811534 by back-splicing (Figure 1c). Additionally, the circular structure of circ_0000519 was validated in H1299, H2170, and A549 cells via Actinomycin D and RNase R analyses. Results showed that the linear GAPDH was decreased by the treatment of Actinomycin D and RNase R, but circ_0000519 was resistant to Actinomycin D and Rnase R (Figure 1d and e, Figure A1). These results suggested that increased circ_0000519 was related to NSCLC.

### 3.2 circ_0000519 overexpression facilitates NSCLC cell growth and metastasis

**Figure 2:**
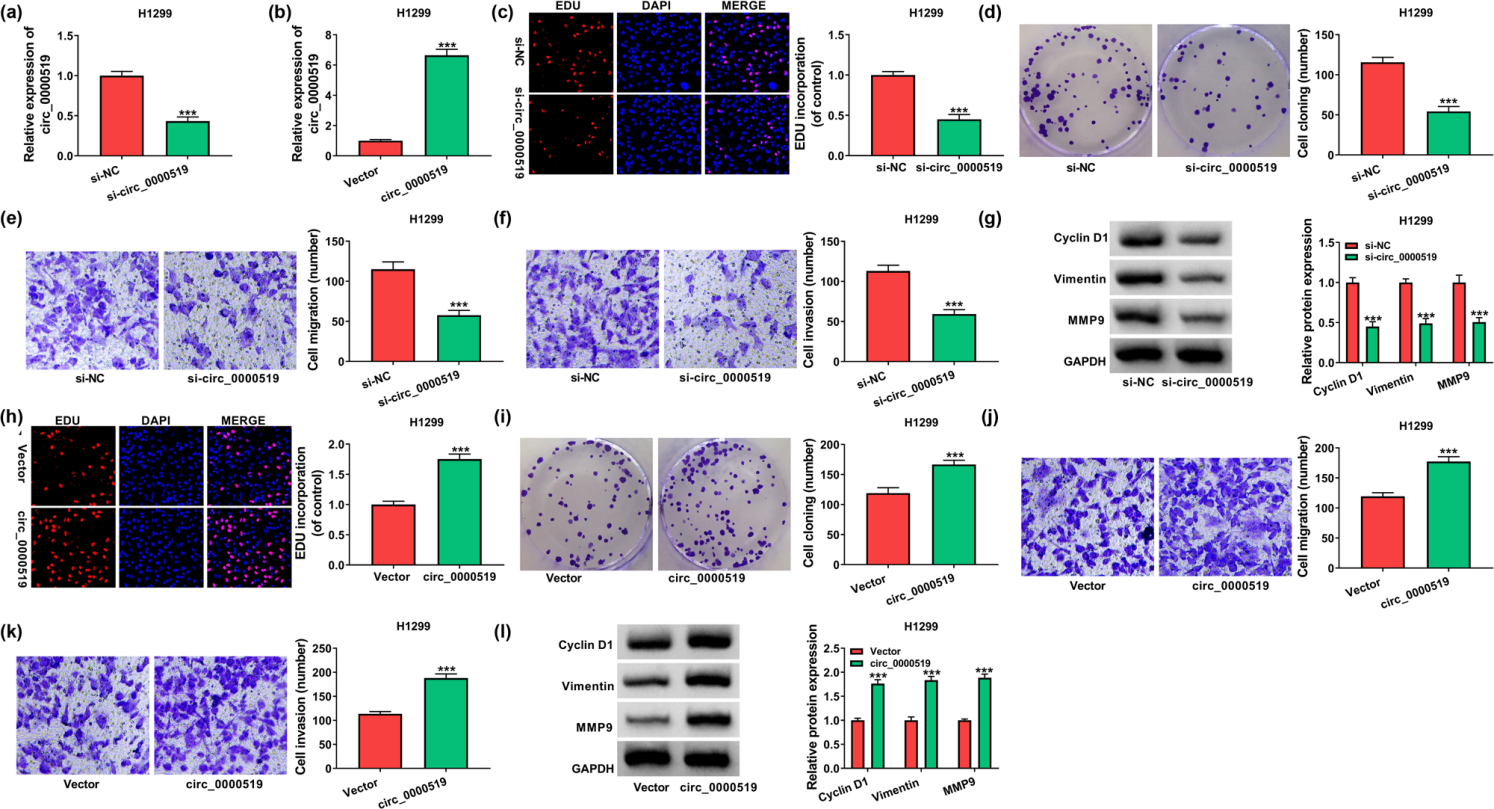
circ_0000519 promotes NSCLC cell growth and metastasis. (a and b) circ_0000519 abundance was examined via qRT-PCR in the H1299 cells with transfection of si-NC, si-circ_0000519, vector or circ_0000519 overexpression vector. (c and d) Cell growth was analyzed using EdU staining and colony formation assays in the H1299 cells transfected with si-NC or si-circ_0000519. (e and f) Cell migration and invasion were measured by a transwell assay in H1299 cells transfected with si-NC or si-circ_0000519. (g) Cyclin D1, Vimentin, and MMP9 levels were measured via Western blotting in the H1299 cells transfected with si-NC or si-circ_0000519. (h and i) Cell growth was detected with EdU staining and colony formation assays in the H1299 cells transfected with vector or circ_0000519 overexpression vector. (j and k) Cell migration and invasion were measured via the transwell analysis in the H1299 cells with transfection of vector or circ_0000519 overexpression vector. (l) Cyclin D1, Vimentin, and MMP9 abundances were determined with Western blotting in the H1299 cells transfected with vector or circ_0000519 overexpression vector. ^***^ *P* < 0.001.

To study the function of circ_0000519 in NSCLC development, H1299 cells were transfected with si-NC, si-circ_0000519, vector or circ_0000519 overexpression vector. The efficacy of si-circ_0000519 or circ_0000519 overexpression vector is validated in Figure 2a and b, which exhibited that circ_0000519 expression was markedly reduced by transfection of si-circ_0000519 and increased by the addition of circ_0000519 overexpression vector. Moreover, circ_0000519 silence evidently decreased cell growth, as revealed by EdU and colony formation assays (Figure 2c and d). Additionally, circ_0000519 interference significantly inhibited cell metastasis by decreasing the migratory and invasive abilities of H1299 cells (Figure 2e and f). Furthermore, the related protein levels were detected, and results showed that circ_0000519 knockdown led to obvious reductions of Cyclin D1, Vimentin, and MMP9 (Figure 2g). In addition, circ_0000519 overexpression increased the EdU-positive cells, colony formation ability, migration, invasion, and the expression of Cyclin D1, Vimentin, and MMP9 (Figure 2h–l). These data indicated that circ_0000519 overexpression contributed to NSCLC cell growth and metastasis.

### 3.3 H1299 cells can release exosomes

**Figure 3:**
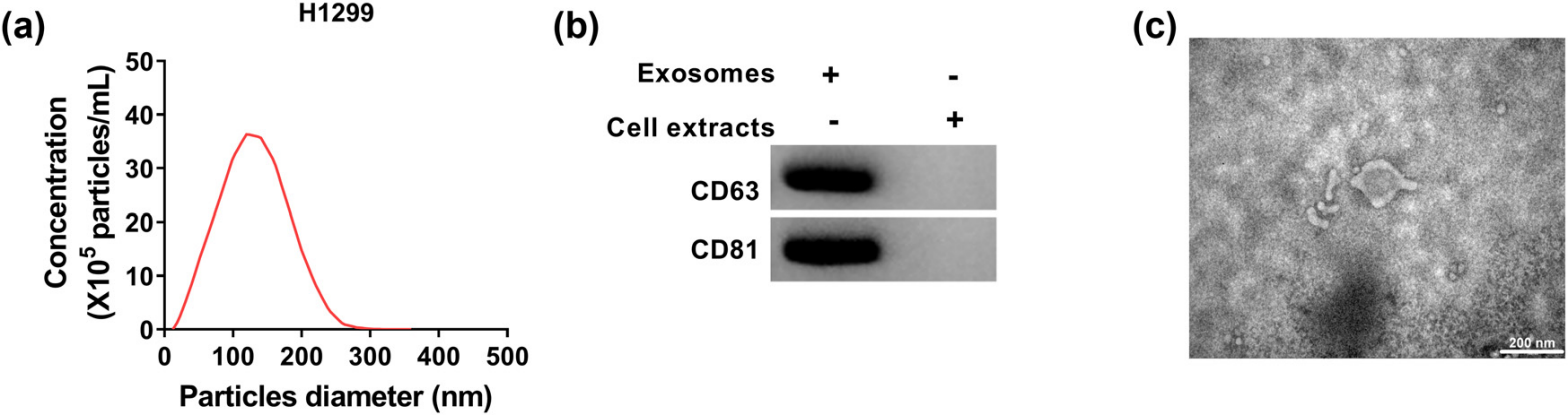
The exosomes are secreted by H1299 cells. Exosomes were isolated from the culture medium of H1299 cells. (a) The exosomes were confirmed by TEM. Scale bar: 200 nm. (b) The diameter of exosomes was analyzed. (c) CD63 and CD81 levels were detected via Western blotting in the exosomes from the culture medium of H1299 cells with or without pre-treatment of 20 μM GW4869 for 2 h.

To explore if circ_0000519 could be transmitted by exosomes, the release of exosomes was analyzed in the H1299 cell medium. The morphology of exosomes was confirmed via TEM, which showed the typical oval-shaped extracellular vesicles (Figure 3a). Moreover, the diameters of exosomes were mainly in the range of 50–200 nm (Figure 3b). Additionally, specific exosomal markers were detected. High levels of CD63 and CD81 were measured in the exosomes from the H1299 cell medium but not detected in the cells with pre-treatment of exosome release inhibitor (GW4869) (Figure 3c). These results suggested that H1299 cells could secrete exosomes.

### 3.4 Exosome-carried circ_0000519 modulates NSCLC cell growth and metastasis

**Figure 4:**
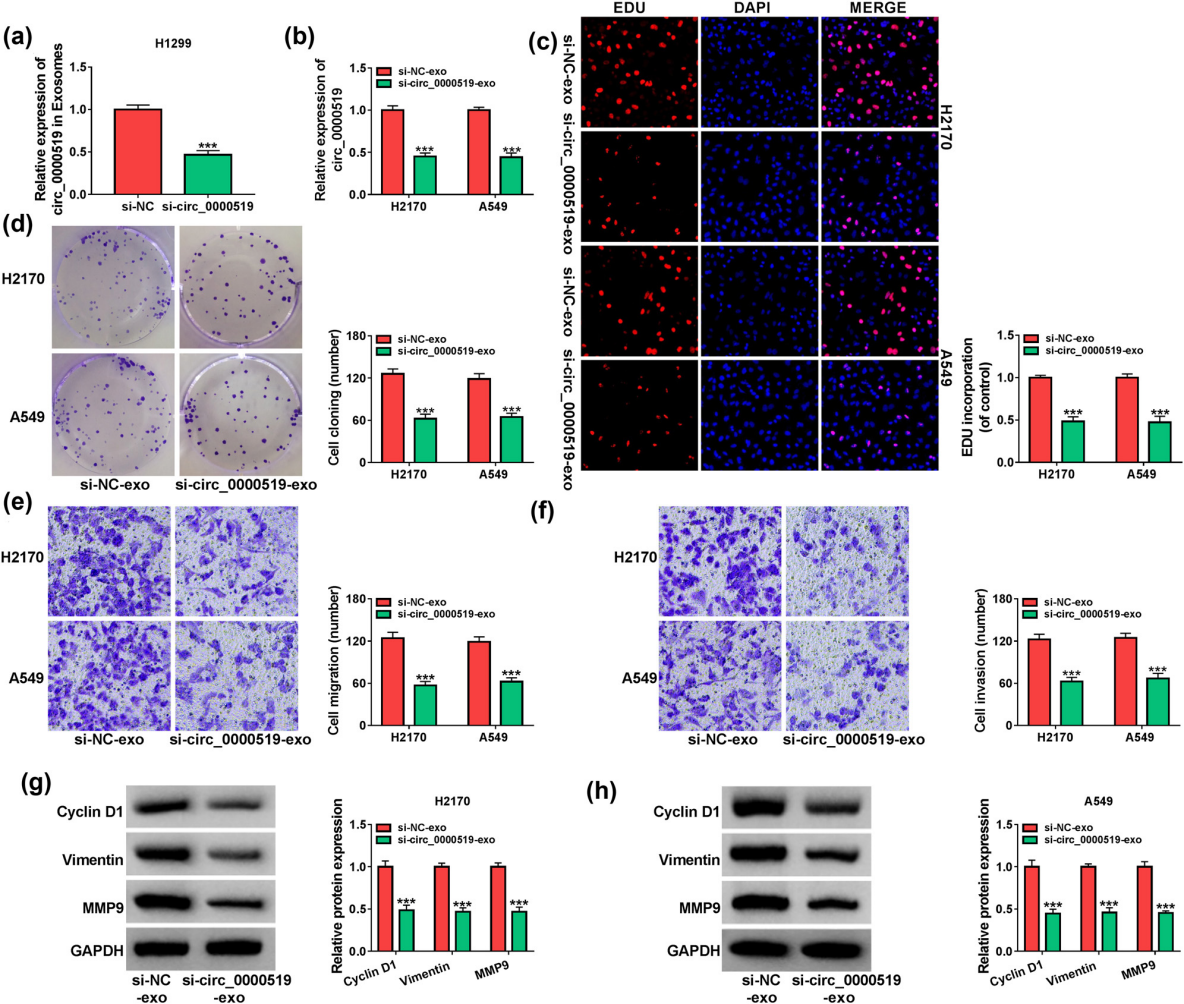
Exosome-carried circ_0000519 regulates NSCLC cell growth and metastasis. (a) circ_0000519 expression was measured using qRT-PCR in the exosomes of H1299 cells transfected with si-NC or si-circ_0000519. The exosomes were isolated from the H1299 cells transfected with si-NC or si-circ_0000519 and then co-incubated with H2170 and A549 cells. (b) circ_0000519 level was measured with qRT-PCR in H2170 and A549 cells after the co-incubation of exosomes. (c and d) Cell growth was analyzed using EdU staining and colony formation assays in H2170 and A549 cells after co-incubation of exosomes. (e and f) Cell migration and invasion were examined through the transwell analysis in H2170 and A549 cells after co-incubation of exosomes. (g and h) Cyclin D1, Vimentin, and MMP9 levels were measured via Western blotting in H2170 and A549 cells after co-incubation of exosomes. ^***^ *P* < 0.001.

The exosomal circ_0000519 expression was markedly decreased in the culture medium of H1299 cells transfected with si-circ_0000519 (Figure 4a). To analyze whether exosomal circ_0000519 could affect the NSCLC development, the exosomes from the medium of si-NC or si-circ_0000519-transfected H1299 cells (named si-NC-exo or si-circ_0000519-exo) were exposed to H2170 and A549 cells. circ_0000519 levels were decreased in the H2170 and A549 cells incubated with si-circ_0000519-exo compared with these cells treated with si-NC-exo (Figure 4b). The EdU-positive cells and colony-formation abilities of H2170 and A549 cells were markedly decreased in the si-circ_0000519-exo-treated group compared with the si-NC-exo-treated group (Figure 4c and d). Additionally, cell migration and invasion were evidently reduced in the H2170 and A549 cells treated with si-circ_0000519-exo in comparison to these cells treated with si-NC-exo (Figure 4e and f). Moreover, the levels of Cyclin D1, Vimentin, and MMP9 were evidently downregulated in the cells treated with si-circ_0000519-exo compared with these cells treated with si-NC-exo (Figure 4g and h). These results suggested that exosomal circ_0000519 could regulate NSCLC cell growth and metastasis.

### 3.5 miR-1258 is sponged by circ_0000519

**Figure 5:**
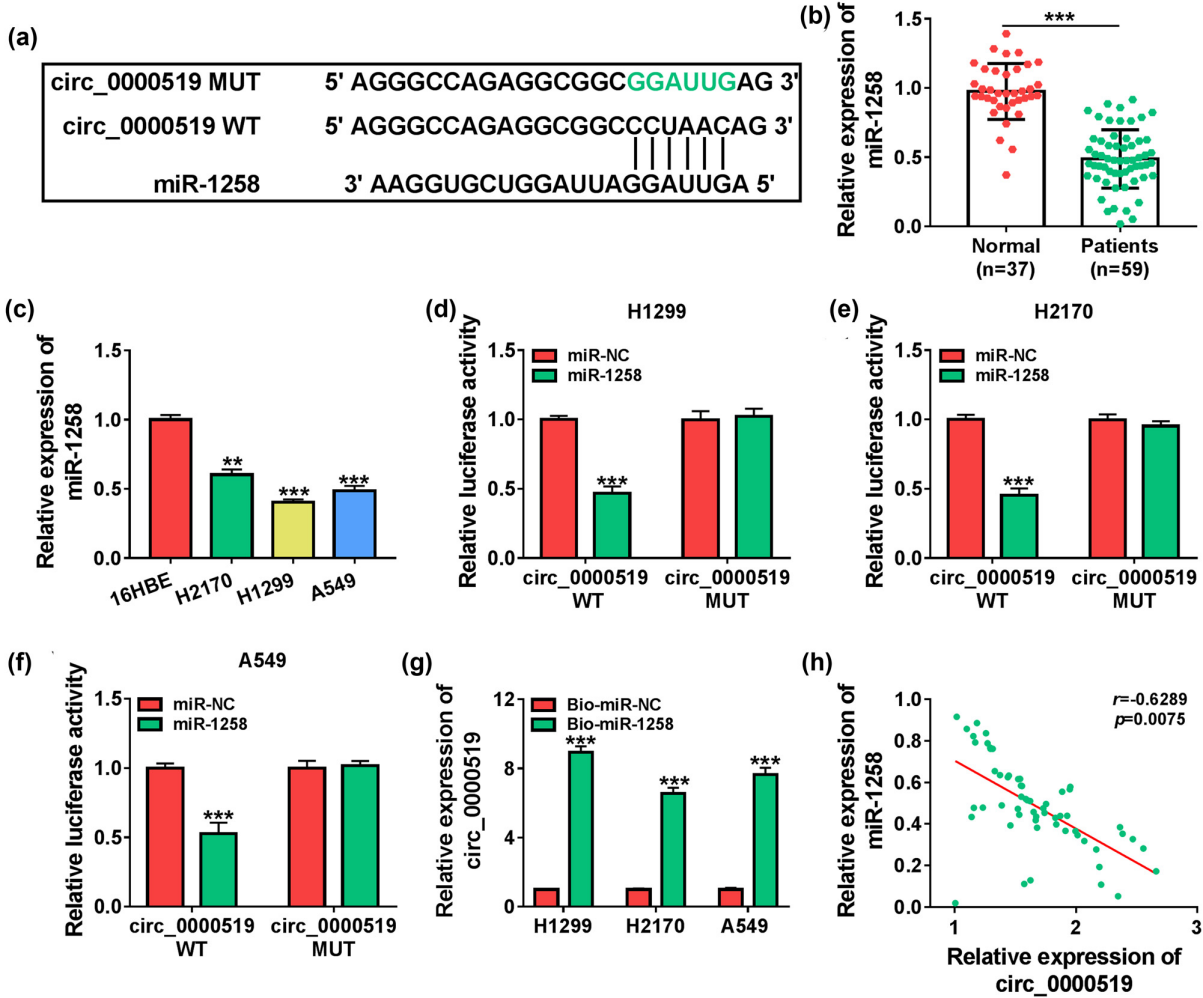
miR-1258 is sponged by circ_0000519. (a) The binding sites of miR-1258 and circ_0000519 were predicted by circinteractome. (b) miR-1258 expression was examined via qRT-PCR in the serum samples of NSCLC patients (*n* = 59) or normal subjects (*n* = 37). (c) miR-1258 abundance was measured via qRT-PCR in H2170, H1299, A549 and BEAS-2B cells. (d–f) Luciferase activity was detected in the H1299, H2170 and A549 cells transfected with circ_0000519 WT or circ_0000519 MUT and miR-NC or miR-1258 mimic. (g) circ_0000519 level was determined via qRT-PCR in cells after RNA pull-down analysis. (h) The linear correlation of miR-1258 and circ_0000519 levels in NSCLC patients. ^***^ *P* < 0.001.

To reveal the potential mechanism modulated by circ_0000519, the downstream miRNAs were predicted by the circinteractome database. The predicted sites of circ_0000519 within miR-1258 are displayed in Figure 5a, suggesting that miR-1258 might be targeted by circ_0000519. miR-1258 expression was decreased in NSCLC patients when compared with normal subjects (Figure 5b). Moreover, the miR-1258 level was markedly reduced in H2170, H1299, and A549 cells when compared with the BEAS-2B cells (Figure 5c). To validate the association of circ_0000519 and miR-1258 in H2170, H1299, and A549 cells, the circ_0000519 WT or circ_0000519 MUT luciferase reporter vectors were constructed. miR-1258 mimic effectively decreased the luciferase activity of circ_0000519 WT but had no significant effect on the activity of circ_0000519 MUT (Figure 5d–f). Furthermore, a high level of circ_0000519 was enriched by Bio-miR-1258 in the three cell lines (Figure 5g). In addition, miR-1258 abundance in NSCLC patients was negatively related to circ_0000519 expression (*r* = −0.6289, *P* = 0.0075) (Figure 5h). These data showed that circ_0000519 could target miR-1258.

### 3.6 RHOV is targeted by miR-1258

**Figure 6:**
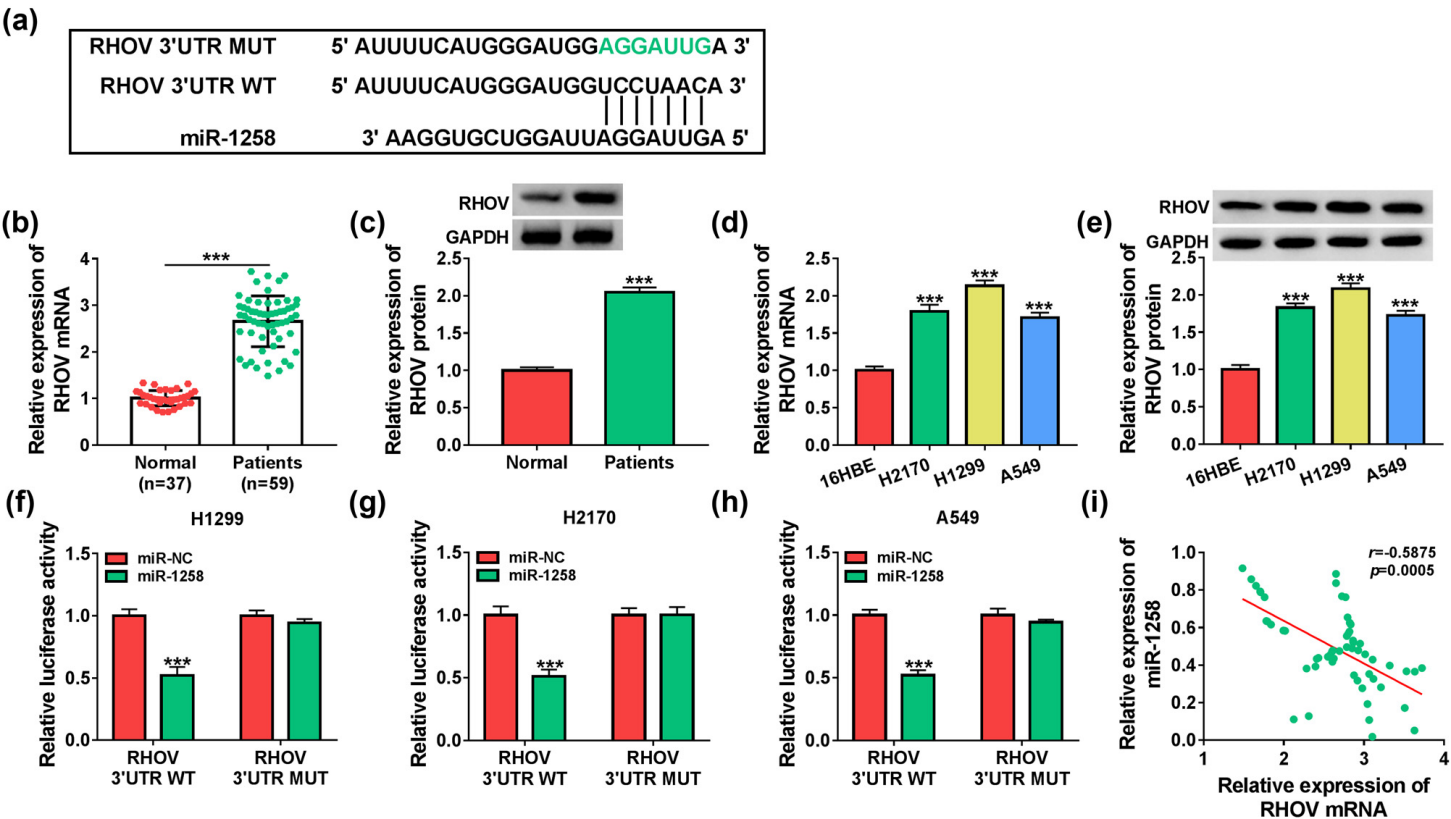
RHOV is targeted via miR-1258. (a) The binding sites of miR-1258 and RHOV were predicted by TargetScan. (b and c) RHOV abundance was detected using qRT-PCR and Western blotting in the serum samples of NSCLC patients or normal subjects. (d and e) RHOV abundance was measured via qRT-PCR and Western blotting in H2170, H1299, A549, and BEAS-2B cells. (f–h) Luciferase activity was detected in the H1299, H2170, and A549 cells transfected with RHOV 3′UTR WT or RHOV 3′UTR MUT and miR-NC or miR-1258 mimic. (i) The linear correlation of miR-1258 and RHOV levels in NSCLC patients. ^***^ *P* < 0.001.

To further explore the downstream of miR-1258, the targets of miR-1258 were predicted by the TargetScan online database. The target sequence of miR-1258 on RHOV is shown in Figure 6a. Moreover, the RHOV expression was significantly enhanced in NSCLC patients (Figure 6b and c). Additionally, the RHOV level was evidently increased in H2170, H1299, and A549 cells when compared with the BEAS-2B cells (Figure 6d and e). To identify the relationship between miR-1258 and RHOV, the RHOV 3′UTR WT and RHOV 3′UTR MUT luciferase reporter vectors were constructed. miR-1258 overexpression caused obvious inhibition of luciferase activity of RHOV 3′UTR WT, while the luciferase activity of RHOV 3′UTR MUT had no response to miR-1258 introduction (Figure 6f–h). Furthermore, miR-1258 level in NSCLC patients was negatively correlated with RHOV expression (*r* = −0.5875, *P* = 0.0005) (Figure 6i). These results showed that miR-1258 could target RHOV.

### 3.7 miR-1258 knockdown reverses the influence of exosomal circ_0000519 downregulation on NSCLC cell growth and metastasis

**Figure 7:**
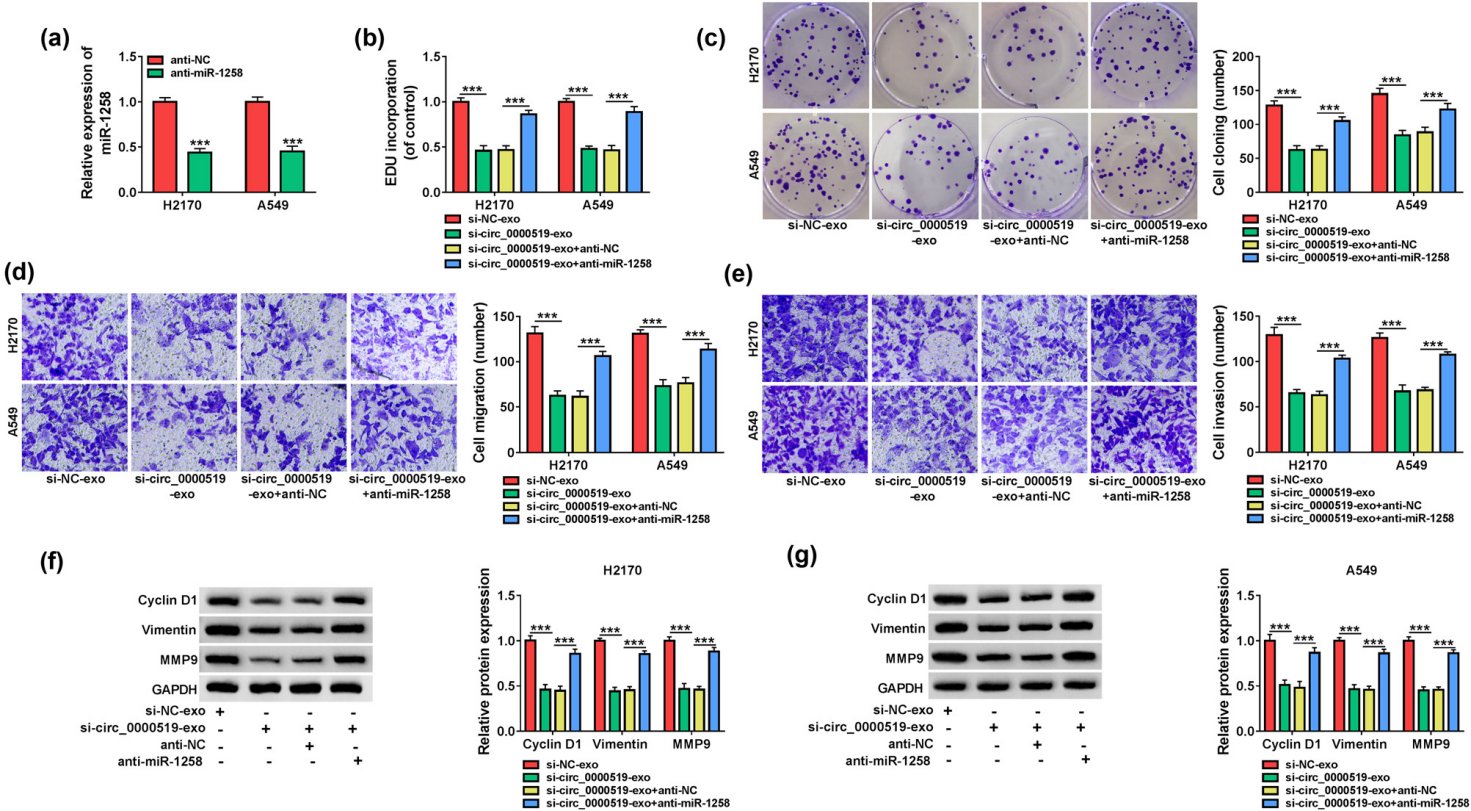
miR-1258 knockdown attenuates the influence of exosomal circ_0000519 downregulation on NSCLC cell growth and metastasis. H2170 and A549 cells were transfected with anti-NC or anti-miR-1258, and then, the transfected or non-transfected cells were incubated with the exosomes from the H1299 cells transfected with si-NC or si-circ_0000519. (a) miR-1258 expression was detected by qRT-PCR in the transfected cells. (b and c) Cell growth was evaluated via EdU staining and colony formation assays in the treated cells. (d and e) Cell migration and invasion were examined via transwell analysis in the treated cells. (f and g) Cyclin D1, Vimentin and MMP9 abundances were examined via Western blotting in the treated cells. ^***^ *P* < 0.001.

To analyze if miR-1258 was associated with exosomal circ_0000519-mediated NSCLC development, H2170 and A549 cells were transfected with anti-NC or anti-miR-1258 and then incubated with the exosomes from si-NC or si-circ_0000519-transfected H1299 cells (named si-NC-exo or si-circ_0000519-exo). The transfection of anti-miR-1258 effectively decreased the miR-1258 abundance in H2170 and A549 cells (Figure 7a). Moreover, miR-1258 knockdown attenuated the si-circ_0000519-exo-mediated inhibition of EdU-positive ratio and colony-formation ability (Figure 7b and c). Furthermore, miR-1258 silence mitigated the suppressive effect of exosomal circ_0000519 reduction on cell migration and invasion (Figure 7d and e). Additionally, miR-1258 knockdown alleviated the decreases of Cyclin D1, Vimentin, and MMP9 levels induced by si-circ_0000519-exo (Figure 7f and g). These data indicated that exosomal circ_0000519 downregulation inhibited the NSCLC cell growth and metastasis via regulating miR-1258.

### 3.8 RHOV upregulation attenuates the influences of exosomal circ_0000519 downregulation on NSCLC cell growth and metastasis

**Figure 8:**
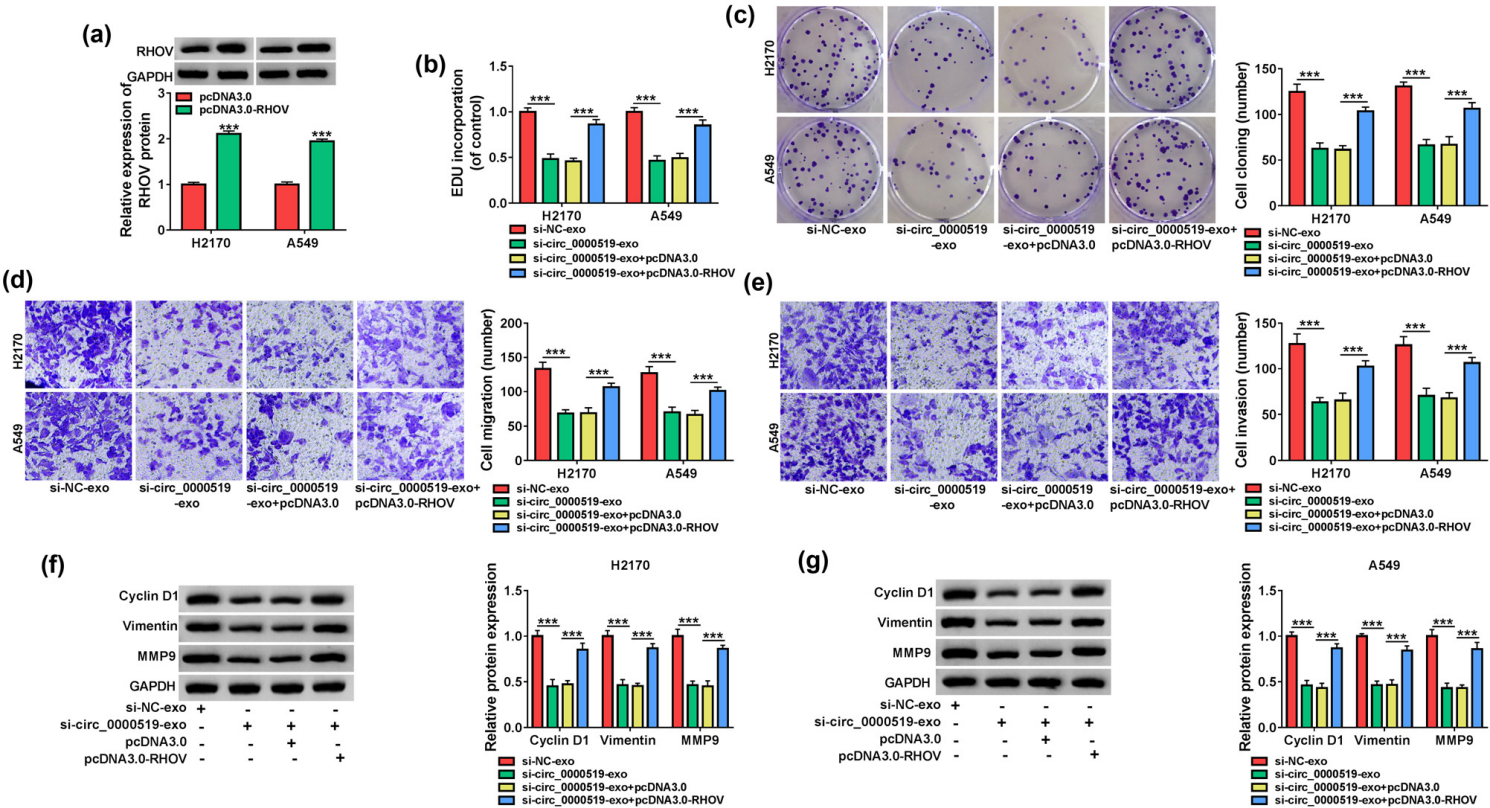
RHOV overexpression relieved the influence of exosomal circ_0000519 downregulation on NSCLC cell growth and metastasis. H2170 and A549 cells were transfected with pcDNA3.1 or pcDNA3.1-RHOV, and then the transfected or non-transfected cells were incubated with the exosomes from H1299 cells transfected with si-NC or si-circ_0000519. (a) RHOV expression was measured via Western blotting in transfected cells. (b and c) Cell growth was analyzed using EdU staining and colony formation assays in the treated cells. (d and e) Cell migration and invasion were examined by a transwell assay in the treated cells. (f and g) Cyclin D1, Vimentin and MMP9 abundances were determined using Western blotting in the treated cells. ^***^ *P* < 0.001.

To analyze if RHOV participated in the exosomal circ_0000519-modulated NSCLC development, H2170 and A549 cells were transfected with pcDNA3.1 or pcDNA3.1-RHOV, and then, the transfected or non-transfected cells were incubated with the exosomes from si-NC or si-circ_0000519-transfected H1299 cells (named as si-NC-exo or si-circ_0000519-exo). The overexpression efficacy of pcDNA3.1-RHOV is confirmed in Figure 8a, which induced a higher level of RHOV. Furthermore, RHOV overexpression attenuated the si-circ_0000519-exo-modulated inhibition of EdU-positive ratio and colony-formation ability (Figure 8b and c). Additionally, RHOV upregulation mitigated the inhibitory effects of exosomal circ_0000519 downregulation on cell migration and invasion (Figure 8d and e). Moreover, RHOV restoration alleviated the decreases of Cyclin D1, Vimentin, and MMP9 levels caused via the addition of si-circ_0000519-exo (Figure 8f and g). These results suggested that the exosomal circ_0000519 downregulation repressed the NSCLC cell growth and metastasis by modulating RHOV.

### 3.9 Exosomal circ_0000519 downregulation reduces xenograft tumor growth

**Figure 9:**
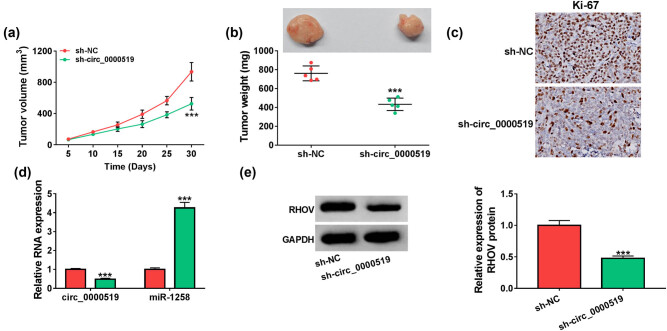
Exosomal circ_0000519 regulates xenograft tumor growth. The murine xenograft model was induced by injection of H2170 cells, and tumors were treated with the exosomes from H1299 cells transfected with sh-NC or sh-circ_0000519 (named sh-NC-exo or sh-circ_0000519-exo) after 5 days. (a and b) Tumor volume and weight were examined in each group. *n* = 5. (c) The Ki-67 level was detected by an immunohistochemistry assay. (d and e) circ_0000519, miR-1258, and RHOV abundances were examined via qRT-PCR and Western blotting in each group. ^***^ *P* < 0.001.

To test the role of exosomal circ_0000519, H2170 cells were subcutaneously injected into nude mice to establish the murine xenograft model; after 5 days, the tumors were treated with the exosomes from H1299 cells transfected with sh-NC or sh-circ_0000519 (named sh-NC-exo or sh-circ_0000519-exo). And, the mice were divided into sh-NC-exo and sh-circ_0000519-exo groups (*n* = 5 per group). As presented in Figure 9a and b, tumor volume and weight were lower in the sh-circ_0000519-exo group than in the sh-NC-exo group. Moreover, the immunohistochemistry results showed that the Ki-67 level was clearly decreased in the sh-circ_0000519-exo group (Figure 9c). Additionally, circ_0000519, miR-1258, and RHOV levels were measured in the tumors. Results showed that circ_0000519 and RHOV levels were markedly decreased, and the miR-1258 level was elevated in the sh-circ_0000519-exo group when compared with the sh-NC-exo group (Figure 9d and e). These data suggested that exosomal circ_0000519 downregulation reduced the NSCLC cell growth in the xenograft model.

## 4 Discussion

NSCLC is the most frequent lung cancer with high mortality worldwide [21]. Although the prevention and detection of NSCLC have gained great advances, the majority of the cases are diagnosed with advanced or metastatic diseases with a poor prognosis [22]. Exosomes are important molecular vehicles that are involved in various processes and are associated with the carcinogenesis and therapy of NSCLC [23]. circRNAs are a type of cargoes delivered via exosomes, which have important roles in cell proliferation, apoptosis, metastasis, and resistance in cancers [24]. Moreover, the circRNA/miRNA/mRNA network is an important mechanism addressed by circRNA [11]. In our research, we first confirmed that exosomal circ_0000519 regulated NSCLC growth and metastasis and found that the inner mechanism was related to the miR-1258/RHOV axis.

According to the GSE101123 dataset, Zhao et al. reported that circ_0000519 was upregulated and was likely associated with tumor development and therapy in breast cancer [25]. Moreover, according to the GSE101684 and GSE101586 datasets, Wang et al. found that circ_0000519 was enhanced in NSCLC and regulated the PDL1/PD1 expression [14]. Similarly, we also confirmed that circ_0000519 was elevated in NSCLC tissues and cells, and we first found the oncogenic function of this circRNA in NSCLC by increasing cell growth and metastasis. Furthermore, Wang et al. also showed that circ_0000519 was secreted by exosomes in NSCLC [14]. The tumor-derived exosomes could take part in the NSCLC development [8], such as circRNA ARHGAP10, circRNA MEMO1, and hsa_circ_0002130 [12,13,26]. Here, we found that circ_0000519 secreted by H1299 cells could affect cell growth and metastasis in H2170 and A549 cells, indicating the tumor-derived exosomal circ_0000519 might promote NSCLC development via regulating tumor cell communication.

Given that circ_0000519 was mostly located in the cytoplasm of NSCLC cells, and the circRNA/miRNA/mRNA networks were the main mechanism for circ_0000519 [14]; here, we wanted to explore another network except for the circ_0000519/miR-34a/PDL1 axis. We first confirmed that circ_0000519 could sponge miR-1258 in NSCLC cells. Zhang et al. reported that miR-1258 could repress cell growth, invasion, and epithelial-to-mesenchymal transition by targeting specific protein 1 (SP1) in oral squamous cell carcinoma [27]. Hwang et al. suggested that miR-1258 could inhibit cell proliferation and migration in colorectal cancer by decreasing cyclin-dependent kinase regulatory subunit 1B (CKS1B) [28]. Moreover, miR-1258 was reported to restrain cell proliferation, migration, and invasion in papillary thyroid cancer via regulating transmembrane protease serine 4 (TMPRSS4) [29]. Additionally, miR-1258 could constrain cell proliferation, migration, and invasion, and regulate stem-like cell properties in breast cancer via regulating lysine demethylases 7A (KDM7A) [30]. These all suggested the anti-growth and anti-metastatic roles of miR-1258 in human tumors. More importantly, Jiang et al. reported that miR-1258 was downregulated in NSCLC and inhibited cell proliferation, migration and angiogenesis by targeting GRB2 [18], indicating the inhibitive influences of miR-1258 on the growth and metastasis of NSCLC. In the present work, miR-1258 was lowly expressed in NSCLC tissues and cells and negatively correlated with circ_0000519 in NSCLC tissues. Further, we found that the exosomal circ_0000519 could affect the NSCLC development by modulating miR-1258.

Next, we explored the downstream of miR-1258 and first confirmed that RHOV was targeted by miR-1258. RHOV is an atypical member of Rho GTPases and plays important roles in the cell cycle, cell adhesion, and migration [31]. A previous study showed that RHOV was associated with tumor growth in prostate cancer [32]. Moreover, RHOV was upregulated in NSCLC, and the NSCLC patients with high expression of RHOV had poor outcomes [19,20]. As described by Zhang et al., RHOV could promote lung cancer cell proliferation, migration, and invasion through the JNK/c-Jun pathway [33]. We also found the increased RHOV in NSCLC tissues and cells, which was also consistent with the data from the Gene Expression Profiling Interactive Analysis (GEPIA; http://gepia.cancer-pku.cn/detail.php). Besides, RHOV expression was negatively correlated with miR-1258 expression in NSCLC tissues. Furthermore, we confirmed that the exosomal circ_0000519 could control the NSCLC development by regulating RHOV. Additionally, we confirmed the role of exosomal circ_0000519 in NSCLC *in vivo*.

In conclusion, the exosomal circ_0000519 downregulation inhibited the cell growth and metastasis in NSCLC, possibly via regulating miR-1258 and RHOV. This research provided a new mechanism for understanding the pathology of NSCLC and identified a new target for the therapy of NSCLC.
